# Secretory pathways and multiple functions of nonstructural protein 1 in flavivirus infection

**DOI:** 10.3389/fimmu.2023.1205002

**Published:** 2023-07-13

**Authors:** Senzhao Zhang, Yu He, Zhen Wu, Mingshu Wang, Renyong Jia, Dekang Zhu, Mafeng Liu, Xinxin Zhao, Qiao Yang, Ying Wu, Shaqiu Zhang, Juan Huang, Xumin Ou, Qun Gao, Di Sun, Ling Zhang, Yanling Yu, Shun Chen, Anchun Cheng

**Affiliations:** ^1^ Research Center of Avian Disease, College of Veterinary Medicine, Sichuan Agricultural University, Chengdu, Sichuan, China; ^2^ Key Laboratory of Animal Disease and Human Health of Sichuan Province, Sichuan Agricultural University, Chengdu, Sichuan, China; ^3^ Engineering Research Center of Southwest Animal Disease Prevention and Control Technology, Ministry of Education of the People’s Republic of China, Chengdu, China

**Keywords:** flavivirus, nonstructural protein 1, structure, secretory pathways, immune evasion, pathogenesis, host-virus interactions

## Abstract

The genus *Flavivirus* contains a wide variety of viruses that cause severe disease in humans, including dengue virus, yellow fever virus, Zika virus, West Nile virus, Japanese encephalitis virus and tick-borne encephalitis virus. Nonstructural protein 1 (NS1) is a glycoprotein that encodes a 352-amino-acid polypeptide and has a molecular weight of 46–55 kDa depending on its glycosylation status. NS1 is highly conserved among multiple flaviviruses and occurs in distinct forms, including a dimeric form within the endoplasmic reticulum, a cell-associated form on the plasma membrane, or a secreted hexameric form (sNS1) trafficked to the extracellular matrix. Intracellular dimeric NS1 interacts with other NSs to participate in viral replication and virion maturation, while extracellular sNS1 plays a critical role in immune evasion, flavivirus pathogenesis and interactions with natural vectors. In this review, we provide an overview of recent research progress on flavivirus NS1, including research on the structural details, the secretory pathways in mammalian and mosquito cells and the multiple functions in viral replication, immune evasion, pathogenesis and interaction with natural hosts, drawing together the previous data to determine the properties of this protein.

## 1 Introduction

The genus *Flavivirus* contains a large number arthropod-borne RNA viruses, many of which can cause severe illnesses in humans, such as dengue virus (DENV), yellow fever virus (YFV), Zika virus (ZIKV), West Nile virus (WNV), Japanese encephalitis virus (JEV) and tick-borne encephalitis virus (TBEV) (1). The distribution and outbreaks of flaviviruses have posed major public health concerns over the past few years. Although the advancements in science that led to the creation of the vaccines and antiviral drugs (2–4), there is still an urgent need for novel anti-flavivirus therapeutics.

**Figure 1:**
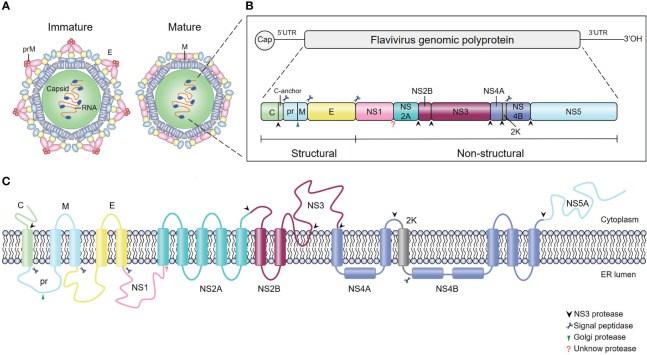
Schematics and structures of flavivirus particles, genome organization and polyprotein. **(A)** The immature (left) and mature (right) forms of flavivirus virions. C, capsid protein; prM, precursor of membrane protein (M); E, envelope protein. **(B)** Schematic representation of flavivirus genome organization and polyprotein. Flavivirus has an approximately 11-kb single-stranded positive-sense RNA genome with an open reading frame (ORF) flanked by the 5’ and 3’ untranslated regions. The single ORF encodes a polyprotein that is subsequently processed into three structural proteins (C, prM and E) and seven nonstructural proteins (NS1, NS2A, NS2B, NS3, NS4A, NS4B, and NS5) by viral and cellular proteases. The blue scissors, black arrows, and green arrows represent cleavage sites for signal peptidase, viral protease, and furin protease, respectively, while the red question mark represents the cleavage site of the unknown protease. **(C)** Schematic of flavivirus polyprotein cleavage. The cylinders represent transmembrane domains of individual proteins, and lines represent transmembrane junctions. Flavivirus polyprotein is cleaved into ten proteins by viral and host cell proteases as described in **(B)**.

Flaviviruses are enveloped, single-stranded, positive-sense RNA viruses with ~50 μm particle size (5) (**Figure 1A**). The viral genome is ~11 kb in length and contains one long open reading frame (ORF) (**Figure 1B**); this ORF encodes a single polyprotein that is cleaved by cellular and viral proteases into three structural proteins (capsid [C], premembrane [prM], and envelope [E]) and seven nonstructural (NS) proteins (NS1, NS2A, NS2B, NS3, NS4A, NS4B, and NS5) (**Figure 1C**). Structural proteins play a critical role in receptor binding, membrane fusion and virion assembly (6), while NS proteins mainly participate in viral RNA replication and innate immune evasion (7–11).

Flavivirus NS1 is an enigmatic protein whose structure and functions still have remained elusive since it was first identified in 1970 (12). Among nonstructural proteins, NS1 is the only one which could be released into extracellular environment (13). Moreover, NS1 is a key factor with multiple roles during flavivirus infection, it directly participates in the formation of the viral replication complex (14–17), is involved in virus maturation (13, 18–20) and mediates viral immune evasion and pathogenesis (8, 21–23). Recently, a study enabled the rapid identification of NS1 mutations that are essential for NS1 secretion and viral replication by combination of the luminescent peptide tagged system with random point mutagenesis (24). In the future, identification of residues that alter NS1 secretion and viral replication may provide some promising strategies for the development of attenuated vaccines.

## 2 The structure and diverse functions of flavivirus NS1

Flavivirus NS1, which was first identified as a soluble complement fixing (SCF) antigen in the serum and brain homogenates of DENV-infected mice in 1970 (12, 25, 26), was subsequently found in the blood of DENV-infected patients (27). At first, this SCF was designated gp48 after its molecular weight was determined by SDS−PAGE (28). Then, when the first flavivirus genome was sequenced in 1985, it was soon renamed NS1 (29).

**Figure 2:**
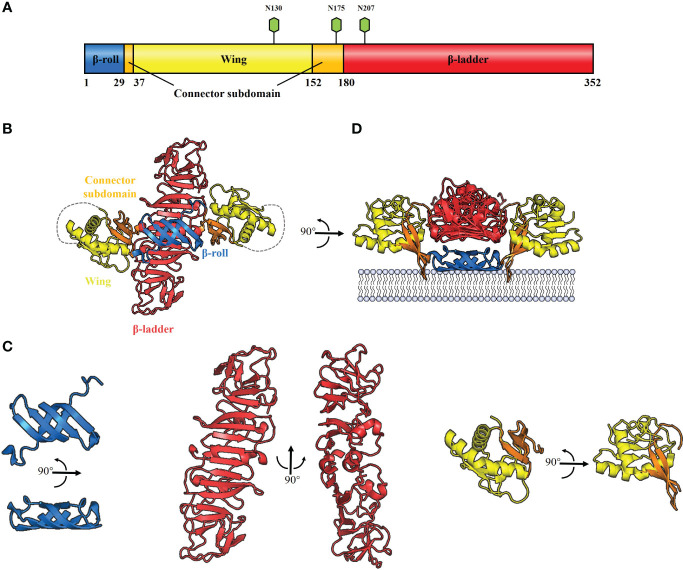
Schematic diagrams of flavivirus NS1 structure. **(A)** The primary structure of flavivirus NS1. The β-roll, wing and β-ladder domains are colored blue, yellow and red, respectively. Two connector subdomains within the wing domain are shown in orange. The glycosylation sites are shown with green hexagons. **(B)** 3D organization of the flavivirus NS1 dimer. The left panel shows a ribbon representation of the 3D crystal structure of the WNV NS1 dimer colored as in A (PDB: 4O6D). A 20-residue disordered region is indicated with gray dotted lines. **(C)** The crystal structure of flavivirus NS1 three domains. The left panel shows the β-roll domain, the center panel shows the β-ladder domain, and the right panel shows the wing domain with the connector subdomain. **(D)** The interaction between hydrophobic protrusion of the NS1 dimer and membrane. Residues that affect virus RNA replication (10–11 and 159–162), were inserted into the membrane surface.

All flavivirus NS1 genes are highly conserved, encoding a 352-amino-acid polypeptide with a molecular weight that varies from 46–55 kDa depending on the glycosylation status (30–33) (**Figure 2A**). NS1 from DENV, ZIKV, YFV and JEV contains two conserved N-linked glycosylation sites, which are found at positions N130 and N207 (34, 35), while a few members of the Flaviviruses, such as WNV, duck Tembusu virus (DTMUV), Murray Valley encephalitis virus (MVEV) and Saint Louis encephalitis virus (SLEV), have an additional site at N175 (36). Intriguingly, except for the glycosylation site at N207, TBEV and Louping ill virus (LIV) have another two glycosylation sites at N85 and N23 (32). The glycosylation of NS1 is crucial for viral replication and virulence (34, 35, 37). For DENV, the mutant virus lacking the glycosylation sites at N207 of NS1 showed reduced growth in cell culture and attenuation of neurovirulence in mice (34). For YFV, lacking the glycosylation sites at N130 or both sites at N130 and N207 of NS1 impaired viral multiplication *in vitro*; in addition, mutant viruses exhibited significant reduction in mouse neurovirulence (35). Moreover, Deglycosylation of DTMUV NS1 also significantly reduced viral multiplication *in vitro* and decreased viral virulence in ducklings (37).

Cryo-electron microscopy analysis of WNV and DENV NS1 provides evidence that each NS1 dimer comprises the following domains (38) (**Figure 2B**): a hydrophobic “β-roll” dimerization domain (amino acids 1-29, **Figure 2C**: left) containing two β hairpins to form a four-stranded β sheet, which curves into a roll-like structure. The second “wing’’ domain (amino acids 38–151, **Figure 2C**: right) has a four-stranded β sheet, two α-helices, and a disordered loop [amino acids 108 to 128 (**Figure 2B**, gray dotted lines)]. Finally, the “β-ladder” domain (amino acids 181-352, **Figure 2C**: center) is formed by 18 continuous β sheets arranged like a ladder. Moreover, the connector subdomain (amino acids 30-37 and 152-180, **Figure 2C**: right) contains a 3-stranded β-sheet that links the wing to β-roll and β-ladder domains.

It has been suggested that the inner face of the NS1 dimer associates with the membrane surface with a “hydrophobic protrusion’’ formed by the residues 10–11 of β-roll and residues 159–162 of the connector subdomain (**Figure 2D**) (38). These residues are important for genome replication, and the same β-roll residues are also implicated in a direct interaction with NS4B (38, 39). These results imply that the hydrophobic protrusion faces the membrane and interacts with other proteins of the replication complex.

## 3 Localization, maturation, and secretion of NS1

### 3.1 Trafficking pathway of NS1

**Figure 3:**
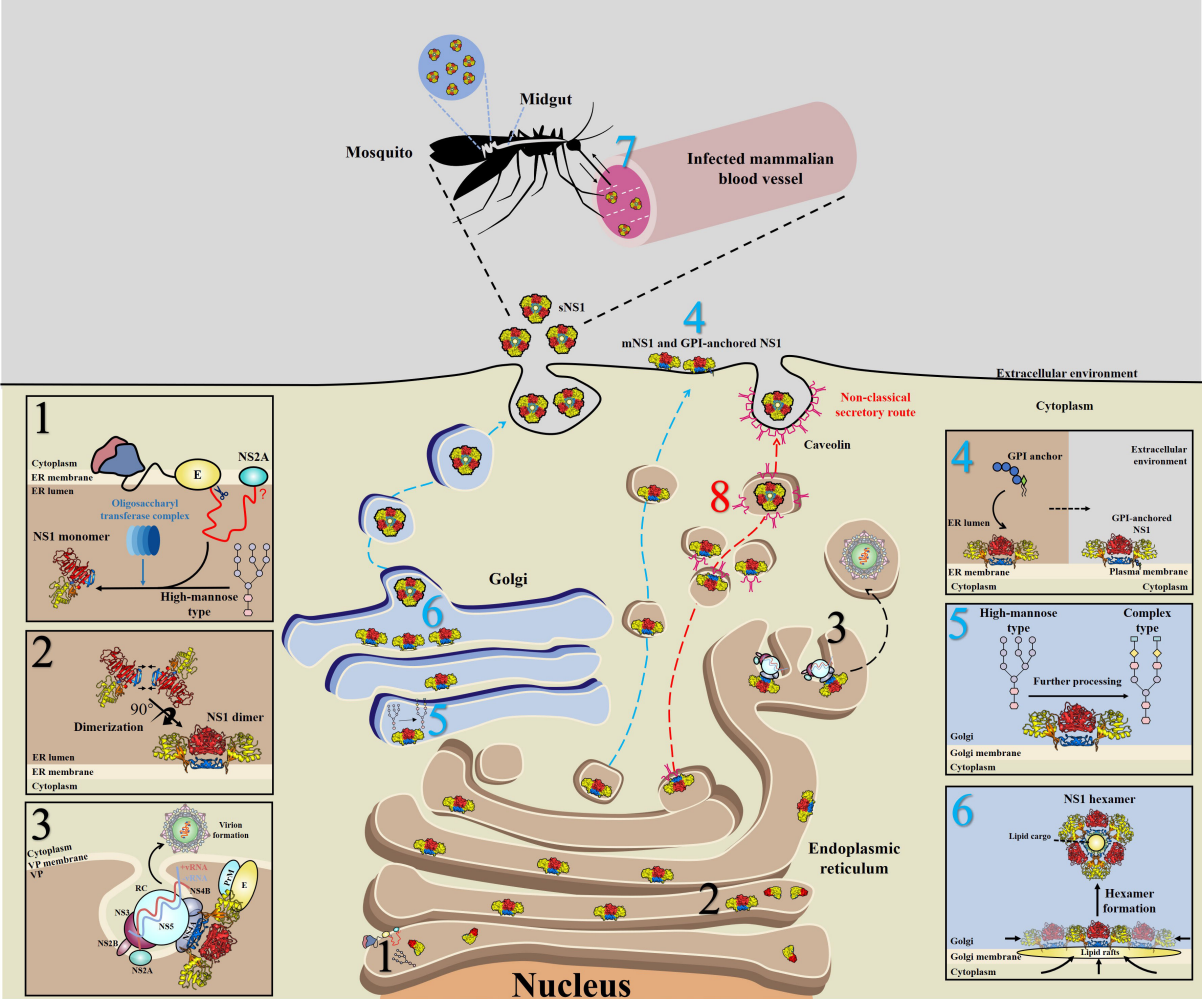
Schematic of NS1 trafficking in mammalian and mosquito cells. 1) During translation, NS1 is trafficked into the endoplasmic reticulum (ER) lumen via a signal peptide at the C-terminus of the E protein and subsequently glycosylated by the addition of high-mannose carbohydrates through the oligosaccharyl transferase complex. 2) Then, NS1 quickly assembles into dimers and exposes a hydrophobic surface, leading to its association with the ER membrane. 3) A part of dimeric NS1 participates in the formation of the viral replication complex (RC) within vesicle packets (VPs). Via interactions with other viral proteins, such as NS4A, NS4B and even prM/E, NS1 links the RC with virion formation. 4) Another part of NS1 is trafficked to the cell surface (mNS1), binding to the cytoplasm membrane via a GPI anchor. 5) The rest of the dimeric NS1 is trafficked to the Golgi for further processing, in which high-mannose glycans are trimmed and more complex glycans are added. 6) Dimeric NS1 assembles into lipidic hexamers (sNS1) and is finally secreted into the extracellular environment. 7) sNS1 is also found in infected mammalian blood, which influences the transmission of virus from hosts to mosquitos by helping virus to overcome the midgut barrier of mosquitos. 8) In insect cells, but not in mammalian cells, sNS1 is secreted to the extracellular media via a nonclassical and caveolin-1-dependent pathway, bypassing the Golgi. Blue, red and black numbers represent that the step occurs in mammalian, insect cells and both two cell lines, respectively.

During translation, the newly synthesized NS1 is translocated into the endoplasmic reticulum (ER) lumen through a signal peptide encoded by the last 24 amino acids of the E protein (40), and the N-terminus of NS1 is cleaved from the E protein by cellular proteases (41); in addition, cleavage between NS1 and NS2A requires the last 8 amino acids (L/M-V-X-S-X-V-X-A) of NS1, although the cellular protease responsible for this cleavage is unknown (42) (**Figure 3**, step 1). The NS1 monomer is modified by the addition of high-mannose-type N-glycosylation profiles with the help of the oligosaccharyl transferase complex (**Figure 3**, step 1) and subsequently forms into dimers (**Figure 3**, step 2), acquiring a hydrophobic surface that may be the major reason why NS1 can associate with the ER membrane (38).

After dimerization, NS1 is divided into three distinct pools: a portion of NS1 interacts with other viral proteins and dsRNA to form the viral replication complex (RC), which indicates that NS1 is an essential protein closely related to viral RNA replication (15, 17, 43, 44) (**Figure 3**, step 3).

In mammalian cells, another part of NS1 is trafficked into the plasma membrane (mNS1) by an unknown traffic route. In addition, a subpopulation of these membrane-associated NS1 proteins could bind to the cell surface through a glycosylphosphatidylinositol (GPI) anchor (GPI-anchored NS1) (45–47) (**Figure 3**, step 4).

In mammalian cells, the remaining NS1 is trafficked to the Golgi for further processing, at which the high-mannose-type glycans are trimmed by glycosidases and processed to more complex carbohydrates by glycosyltransferases (48–50) (**Figure 3**, step 5), forming a soluble hexamer (sNS1) that is secreted into the extracellular environment (48–50) (**Figure 3**, step 6). Furthermore, it has been found that sNS1 could bind back to the plasma membrane from the supernatant via an interaction with glycosaminoglycans (GAG), heparin sulfate (HS) and chondroitin sulfate E (51–53). Moreover, sNS1 was also found in the serum of infected hosts (54, 55) and could be acquired by mosquitos along with infecting virions (**Figure 3**, step 7). Additionally, the role of sNS1 inside the mosquito must be further identified.

### 3.2 Differences in the location and secretory route of NS1 in vertebrate and mosquito cells

Differences were observed regarding the location of NS1 between vertebrate and mosquito cells. It has been pointed out that mNS1 can located on the face of the plasma membrane in vertebrate cells (45–47). However, new evidence has demonstrated that despite the high level of sNS1 is detected in the supernatant, mNS1 is not present on the surface of infected mosquito cells by using a commercial ELISA, confocal microscopy, immunoelectron microscopy and cell cytometry assays (56), indicating that there is a reciprocal relationship between sNS1 and mNS1.

Moreover, since sequence analysis showed that flavivirus NS1 is a hydrophilic protein that lacks a transmembrane domain (29), it is intriguing to find that GPI-anchored NS1 is targeted to the cell surface, in which it is lipid raft associated (47). The hydrophobic domain in the N-terminus of NS2A acts as a signal sequence for GPI linkage of NS1, and this specific posttranslational modification occurs in the ER following the cleavage of the signal (47). This study is the first report to identify the linkage between GPI and NS1, but only for DENV. In addition, lower levels of GPI-linked NS1 are expressed in mosquito cells than in vertebrate cells (47). Thus, the inefficient addition of a GPI anchor in the NS1 protein produced in mosquito cells suggests that NS1 plays different roles in vertebrate and insect hosts. Further studies are needed to clarify why the addition of GPI to NS1 is inefficient in mosquito cells.

In addition, for decades, it was widely accepted that flavivirus NS1 was secreted only from infected vertebrate cells rather than from mosquito cells (13, 57, 58). The notion is initially based on observations that the JEV-infected three mosquito cell lines, including C6/36, AP61 and TRA284 cells, did not produce sNS1, while it was detected in the supernatant of infected Vero cells (13). But by using a commercial ELISA, confocal microscopy, immunoelectron microscopy and cell cytometry assays, new evidence challenges this notion and showed that mosquito cells also secrete NS1 efficiently (56, 59, 60). NS1 was detected in supernatants from DENV2- or DENV4-infected mosquito cells starting at 6 hpi, and the concentration of sNS1 increased steadily up to 24 hpi; in addition, the cell viability was evaluated to be over 96% in all cases, suggesting that the presence of NS1 was due to true secretion rather than cell lysis (56). NS1 was also detected in the supernatants of YFV-infected mosquito C6/36 cells using a quantitative NS1-capture ELISA (59), and recombinant ZIKV NS1 was expressed in insect *Drosophila* S2 cells (60). These observations suggest that flaviviruses possess the capacity to secrete NS1 from insect cells.

Further studies found differences between vertebrate and mosquito cells in the secretory route of sNS1 (61). In vertebrate cells, the classical NS1 secretion pathway includes progression from the ER to the Golgi (**Figure 3**, step 4, 5 and 6). In addition, virion release into the extracellular space occurs by this pathway in both vertebrate and mosquito cells. In this study, brefeldin A (BFA) was used to disrupt the function of the trans-Golgi network (TGN) of DENV-infected vertebrate and mosquito cells. As expected, drug treatment of infected vertebrate cells resulted in significantly decreased virions and sNS1. However, interestingly, the secretion of virions was decreased significantly while the secretion of sNS1 remained unchanged in BFA-treated mosquito cells. However, when gene silencing of SAR1 protein expression was performed to disrupt the classical secretory pathway in mosquito cells, the secretion of sNS1 was not inhibited but virion release was significantly reduced. In contrast, gene silencing of caveolin 1 (CAV1), a component of caveolae and a protein that participates in cholesterol transport, resulted in the inefficient secretion of sNS1 without inhibiting virion release. The interaction between NS1 and CAV1 in infected mosquito cells was also verified (61). Recently, it was clearly demonstrated that sNS1 is secreted from mosquito cells in association with the chaperone caveolin complex, a complex formed by CAV-1 and the chaperones FKBP52, Cy40, and CyA (62). Based on these findings, it is reasonable to speculate that sNS1 is secreted from infected mosquito cells via a nonclassical and caveolin-1-dependent pathway that bypasses the TGN (**Figure 3**, step 8).

sNS1 produced in mosquito cells lacks a GPI anchor, which should be involved in trafficking proteins from the ER to the cell surface via the Golgi (63, 64), which may reasonably explain the nonclassical secretory route of NS1 in mosquito cells. Different secretory routes of NS1 may contribute to different structures or modifications that may be connected to the specific effects of extracellular NS1, such as transmission to the next host. Thus, it is necessary to fully dissect the route of secretion of NS1 in mosquito cells.

### 3.3 Differences in NS1 glycosylation between vertebrate and mosquito cells

There are also differences in the glycosylation modification machinery of flavivirus NS1 between vertebrate and insect cells. NS1 is modified by the addition of high mannose carbohydrates in the ER, and then its secreted form is trafficked to the trans-Golgi network for more complex glycan processing in vertebrate cells. In contrast to those of vertebrate cells, glycoproteins in insect cells contain only mannose residues (65). In these cells, as mentioned above, NS1 accumulates instead of being secreted, which suggests that an interaction could occur between secretion and the addition of complex carbohydrates (13, 50). Thus, initially, the inability of insect cells to process NS1 into the complex N-linked glycan form was thought to be responsible for the inefficient secretion of NS1 (13, 65).

Previous studies have demonstrated that the secretion of NS1 in vertebrate cells requires the presence of N-linked glycosylation sites (66), and this requirement was the same in insect cells; specifically, treatment with tunicamycin (an inhibitor of N-linked glycosylation that blocks glycosidase activity) led to a decreasing number of sNS1 in the supernatants (67). In fact, high-mannose glycans at N207 were thought to stabilize the NS1 dimer, as the NS1 dimer of N207A became more temperature sensitive (50). In addition, ineffective secretion of N207Q and N130Q/N207Q NS1 was observed in infected cells (68), which may be explained by the inefficient secretion of sNS1 that occurred due to the misfolding of dimer NS1. Similarly, complex glycans at N130 are critical for stabilizing the secreted hexamer (68), as N130Q and N130/N207Q NS1 showed less hexamer formation than that of WT and N207Q NS1. Moreover, compared to sNS1 from mammalian cells, sNS1 from mosquito cells is less stable and more heat sensitive (67), which is in agreement with the notion that the addition of complex N-glycans at N130 is important but not essential for NS1 secretion (66).

Moreover, mammalian cell-derived NS1 exhibits both high mannose and complex N-glycosylation profiles, which can only be removed by peptide N glycosidase F (PNGase F) (cleaves all N-linked carbohydrates, including high mannose, hybrid and complex glycans) treatment but is resistant to endoglycosidase H (Endo H) treatment (only removes high mannose profiles); however, sNS1 from DENV-infected insect cells not only can be digested with PNGase F but is also sensitive to Endo H treatment, although another viral E glycoprotein is resistant to Endo H treatment. These results also indicated that in mosquito cells, the virions were released through the Golgi, while sNS1 could be secreted into the supernatant though a different secretory pathway that could bypass the Golgi.

## 4 Role of NS1 in viral replication

In the early years of flavivirus research, NS1 was considered to participate in the assembly and release of flavivirus particles due to its localization in the ER lumen (13, 18, 19). However, a finding demonstrating the colocalization of NS1 with viral dsRNA (15) is inconsistent with the postulated role of NS1 in virion assembly and/or maturation. Since this initial finding suggested a possible role for NS1 in RNA replication, an increasing number of trans-complementation experiments have revealed that flavivirus NS1 is involved in the early stage of RNA replication (14, 16, 17, 69).

For example, an NS1-defective YFV or WNV (Kunjun) genome could recover its viral replication by homologous helper NS1 supplied in *trans (* 17, 43). Interestingly, this trans-complementation of NS1 was species specific, as DENV NS1 could not complement an NS1-deficient YFV genome (44). However, a suppressor mutant (N42Y) in NS4A was found to facilitate the ability of NS1-deleted virus to utilize DENV NS1. This study first reveals a genetic interaction between NS1 and NS4A and shows the significance of this interaction for RNA replication.

In addition, a finding clearly provided evidence of a genetic interaction between NS1 and NS4B (39), as substitution of two amino acids at positions 10 and 11 from DENV with WNV NS1 (RQ10NK) facilitated surface expression and secretion of NS1 yet impaired viral replication; however, a mutation (F86C) in NS4B rescued the attenuated RNA replication of mutant virus. Further supportive evidence was obtained using mass spectrometry and coimmunoprecipitation, suggesting a physical interaction between NS1 and NS4B (39). As NS4A and NS4B are both transmembrane viral proteins that span the ER membrane, a reasonable hypothesis is that NS1 performs its diverse functions for the RC via an interaction with NS4A and/or NS4B within the ER lumen.

In addition to interaction with NS4A and/or NS4B to participate in the early stage of viral RNA replication, new evidence has demonstrated that NS1 interacts with the structural proteins prM/E within the ER lumen, assists membrane bending and envelopment of nucleocapsids, and eventually mediates the production of infectious virus particles (20).

Additionally, changes in disulfide bonds or glycosylation sites on the NS1 protein also affect viral replication (35, 37, 70). The newest research demonstrated that deglycosylation of TMUV NS1 impaired viral multiplication *in vitro*, especially in avian cells (37). For YFV, elimination of the first glycosylation site in NS1 also reduced the viral titers (35). Moreover, three mutants at Cys sites in DENV NS1 abolished viral growth, which suggests that the conserved disulfide bond is necessary for viral replication.

Clearly, many uncertainties still exist regarding the interactions between NS1 and other viral proteins, and further research will provide promising advice for antiviral therapies.

## 5 Multifunction of sNS1 *in vivo*

While the ER-resident dimer NS1 is involved in the formation of viral RC, the hexamer sNS1 is responsible for flavivirus pathogenesis and tissue specificity. The circulation of sNS1 in sera could be detected in flavivirus-infected animals and humans (27, 71). Because high levels of sNS1 (up to 10 μg/ml) are present early during viral infection, sNS1 has been identified as a diagnostic marker (58, 72). In addition, although the functions of sNS1 in the virus life cycle have been less fully characterized, new evidence has demonstrated that sNS1 can also participate in the formation of infectious particles by flaviviruses through interactions with the lipid membrane (73, 74).

**Figure 4:**
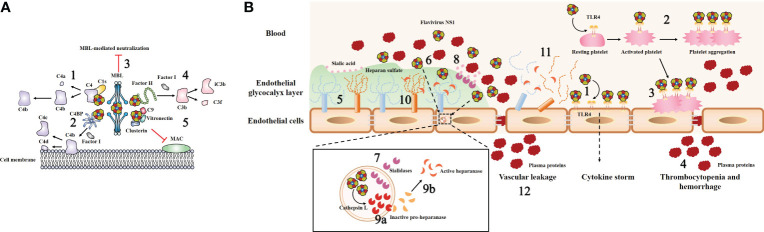
Model of flavivirus sNS1-induced complement evasion and hyperpermeability of endothelial cells. **(A)** Flavivirus sNS1 was shown to involve in viral immune evasion through interaction with complement system components. sNS1 binds to proC1s/C1s and C4, forming a complex to promote efficient degradation of C4 to C4a and C4b, thus limiting the amount of C4 available (1). sNS1 also binds and recruits the complement regulatory protein C4 binding protein (C4BP) to the cell surface. Once bound to NS1, C4BP can act as a cofactor for Factor I-mediated cleavage of C4b to C4c and C4d (2). In addition, DENV sNS1 binds to human mannose-binding lectin (MBL) and protects DENV from MBL-mediated neutralization (3). WNV sNS1 recruits the alternative complement pathway regulatory protein factor H to activate Factor I-mediated cleavage of C3b to iC3b and C3f, thus attenuates C3b and C5b–9 deposition on cell surfaces (4). sNS1 also inhibits the formation of the membrane attack complex (MAC) directly by interacting with clusterin, vitronectin, and the terminal complement protein C9 (5). **(B)** Flavivirus sNS1 has also been shown to induce hyperpermeability in endothelial cells. Flavivirus sNS1 can trigger TLR4 signaling, leading to the secretion of pro-inflammatory cytokines (1). Moreover, sNS1 can bind to platelets via TLR4, leading to platelet activation and enhancement of the platelet aggregation (2). Then the activated platelets are adhered to the cell surface (3) and cause thrombocytopenia and hemorrhage (4). The endothelial glycocalyx layer (EGL) is a network of membrane-bound proteoglycans and glycoproteins that lines the endothelium on the luminal side (5). Flavivirus sNS1 binds to the surface of endothelial cells (6), upregulates the expression of sialidases (7) that translocate to the cell membrane, initiating the cleavage of sialic acid on EGL (8). In addition, flavivirus sNS1 enhances the activity of cathepsin L and the expression of pro-heparinase (9a). Cathepsin L processes pro-heparanase into an active form (9b), leading to the cleavage of heparan sulfate on the EGL (10). These processes together lead to EGL disruption of endothelial cells (11) and vascular leakage during flavivirus infection (12).

Recently, it was clearly demonstrated that extracellular sNS1 functions in the evasion of the immune response and mechanisms of pathogenesis by interacting with host cell regulatory proteins, such as complement system components, the endothelial glycocalyx layer (EGL) and Toll-like receptors (**Figure 4**). Moreover, sNS1 also facilitates flavivirus infection by modulating mosquito antiviral mechanisms.

### 5.1 sNS1 and complement system

The complement system is a central component of the innate immune response and comprises one of the first lines of host defense against pathogens (75). It can be rapidly activated by pathogens via the following pathways: the classical, lectin, or alternative pathways. A previous study found a link between flavivirus NS1, complement activation and disease severity, as both mNS1 and sNS1 could drive complement activation in the presence of antibodies; as a result, anaphylatoxins and soluble terminal complement complex SC5b-9 were generated, which may contribute to vascular leakage during DENV infection (76).

However, to counteract this host’s immune response, viruses have evolved a series of strategies to prevent complement activation and neutralization (77). Since flavivirus NS1 was first described as an SCF antigen in 1970, the immune evasion activity of this protein has been slowly elucidated; flavivirus NS1 could antagonize complement activation via different pathways by interacting with the complement system components, thus limiting inhibitory effects during flavivirus infection (21, 67, 78, 79).

Attenuation of the classical and lectin pathways of complement activation by NS1 is promoted by the following three routes: sNS1 binds to proC1s/C1s and C4, forming a complex to promote efficient degradation of C4 to C4b, thus limiting the amount of C4 available (**Figure 4A**, step 1) (78). Moreover, sNS1 from DENV, WNV and YFV binds and recruits the complement regulatory protein C4 binding protein (C4BP) to the cell surface. Once bound to NS1, C4BP can act as a cofactor for Factor I-mediated cleavage of C4b in solution (**Figure 4A**, step 2) (79). In addition, deposition of the sNS1-C4BP complex on the cell membrane could also inactivate membrane-bound C4b, thus protecting viruses and infected cells from complement attack (79). In addition, DENV NS1 has been shown to bind to human mannose-binding lectin (MBL), thereby protecting DENV against MBL-mediated neutralization (**Figure 4A**, step 3) (67).

Another immune evasion function of sNS1 was verified by the observation that WNV NS1 binds to an alternative complement pathway regulatory protein, factor H (fH); this results in attenuated complement activation as well as reduced deposition of C3 fragments and formation of C5b-9 membrane attack complexes (**Figure 4A**, step 4) (21). Notably, this is not a universal phenomenon among flavivirus NS1, as reports have stated that DENV and JEV NS1 do not bind to fH (67, 80). Thus, it is reasonable to speculate that although the antagonism of complement pathways by NS1 seems to be a common strategy employed by flaviviruses, the potential role of NS1 in flavivirus pathogenesis varies in different flaviviruses, and there may be some evolutionary adaptations that are unique to specific virus−host interactions.

Moreover, a previous study showed that DENV sNS1 could also inhibit the terminal complement pathway by interacting with clusterin, a complement inhibitory factor that inhibits the formation of the membrane attack complex (MAC) (81). Recently, a novel DENV sNS1 binding partner, vitronectin (VN), was identified (82). DENV sNS1, either by itself or in association with VN, interferes with terminal complement protein C9 polymerization and, consequently, MAC formation (**Figure 4A**, step 5) (82). These data imply that NS1 plays a direct role as an inhibitor of the terminal complement pathway.

Notably, there is a conserved complement control protein (CCP) domain within the NS1-binding complement partners, including C1s, C4 and fH. In addition, it should be noted that the complement-binding partners from other pathogens could interact with the CCP domain via an anti-parallel β-sheet motif (83–85). Thus, flavivirus NS1 are likely recognized by those complement proteins due to the high structural homology between the β-ladder domain of NS1 and this motif. Further experiments are necessary to fully dissect regions within NS1 that are involved in the regulation of the complement pathway. However, it is important to examine the multifaceted relationship between NS1 and the complement system more deeply, which will provide some new insights into the effects of flavivirus NS1 on viral pathogenesis and provide a novel target for anti-flavivirus therapies and vaccine development.

### 5.2 sNS1 and endothelial glycocalyx layer disruption

Vascular hyperpermeability is a hallmark of endothelial barrier dysfunction, which can lead to more fluid molecules and biomacromolecules passing through endothelial cells. It has been clearly demonstrated that DENV NS1 can activate Toll-like receptor 4 (TLR4) on mouse macrophages and human peripheral blood mononuclear cells, leading to a “cytokine storm” secretion of pro-inflammatory cytokines (e.g., TNF-α, IL-6, IL-1β and IL-12) that can result in vascular leak (**Figure 4B**, step 1) (86). Further study revealed that DENV NS1 could activate platelets by directly binding to TLR4 on their surfaces and subsequently enhancing platelet aggregation (**Figure 4B**, step 2) (87). Then, the activated platelets tend to adhere to endothelial cells (**Figure 4B**, step 3) or undergo phagocytosis by macrophages, leading to vascular leakage and thrombocytopenia during DENV infection (**Figure 4B**, step 4) (87). The new evidence subsequently demonstrated that only full-length NS1 was able to induce a stable platelet aggregation and the wing domain of NS1 alone is the only one that can also induce platelet activation by itself (88). Furthermore, another study found that inoculation of mice with DENV NS1 results in increased vascular leakage and induction of pro-inflammatory cytokines (e.g., TNF-α, IL-6), while a sublethal DENV inoculum combined with NS1 leads to a lethal vascular leak syndrome (23). However, on the one hand, anti-NS1 monoclonal antibodies or NS1 polyclonal mouse sera could effectively prevent increased leakage *in vitro* and mortality in mice (23). On the other hand, immunization with recombinant NS1 also protected mice against lethal DENV challenge *in vivo (* 23). In addition, a recent work has demonstrated that DENV NS1 could disrupt barrier integrity in primary human endothelial cells by activating the p38 MAPK pathway (89). Taken together, these observations suggested that DENV NS1 can trigger endothelial hyperpermeability, resulting in vascular leakage (23, 86, 89). However, there are still many unknowns concerning the mechanism by which NS1 induces hyperpermeability directly in endothelial cells.

Previous studies have noted that the key factors of endothelial barrier function are tight and adherens junctions (90). However, over the past few years, the endothelial glycocalyx layer (EGL) has also been identified as a potential regulator of vascular permeability (91). The EGL is a network of membrane-bound proteoglycans and glycoproteins that lines the endothelium on the luminal side (**Figure 4B**, step 5) (92). It contains acidic oligosaccharides and terminal sialic acid (N-acetyl-neuraminic acid, Sia), as well as GAG side chains, including HS, hyaluronic acid, and chondroitin sulfate (92). The integrity of the EGL *in vitro* was disrupted by DENV serotypes 1–4 but not the related WNV NS1 (51). DENV NS1 could binds to the surface of endothelial cells (**Figure 4B**, step 6) and increases the expression of cellular enzymes sialidases (**Figure 4B**, step 7), resulting in degradation of Sia (**Figure 4B**, step 8) (51). DENV NS1 also increases the activity of cathepsin L and the expression of pro-heparinase (**Figure 4B**, step 9a). Then cathepsin L processes pro-heparanase into mature form heparinase (**Figure 4B**, step 9b), leading to cleavage of HS chains on the EGL (**Figure 4B**, step 10) (51), and these processes together lead to EGL disruption of endothelial cells, (**Figure 4B**, step 11) and vascular leakage during flavivirus infection (**Figure 4B**, step 12). Inhibitors of these enzymes block DENV NS1-triggered EGL disruption and endothelial hyperpermeability (51). These data suggest a novel role for DENV NS1 in triggering EGL disruption and hyperpermeability during dengue infection. The potential relationship between “cytokine storm” and DENV NS1-mediated vascular leakage was subsequently explored (93). Interestingly, human dermal endothelial cells did not produce inflammatory cytokines (e.g., IL-6, TNF-α, or IL-8) when exposed to DENV NS1 *in vitro (* 93).

Furthermore, it was found that DENV NS1-triggered vascular leak was independent of inflammatory signaling by using TLR4- or TNF-α receptor-deficient mouse models (93). Blocking molecules participating in EGL disruption reduced DENV NS1-induced vascular hyperpermeability (93). Altogether, these results indicated that the disruption of EGL but not the expression of inflammatory cytokines is essential for DENV NS1-triggered vascular leakage *in vitro (* 93).

Moreover, a finding further identified the key molecules in DENV NS1-induced vascular leakage, including endothelial hyperpermeability-related factor macrophage migration inhibitory factor (MIF), glycocalyx degradation-related molecules heparanase 1 (HPA-1) and metalloproteinase 9 (MMP-9) (94). These results illustrated that DENV NS1 induced EGL degradation and hyperpermeability by activating HPA-1 and MMP-9 in an MIF-dependent manner (94).

Recently, the cellular upstream pathway engaged in NS1-induced EGL disruption was illustrated (52). A single glycosylation site mutant (N207Q) inhibits the disruption of endothelial barrier function induced by DENV NS1. The N207Q mutant bound to endothelial cells comparably to WT NS1 but could not be internalized by endothelial cells. Further study showed that NS1 was endocytosed into cells in a clathrin-dependent manner, which is necessary to trigger endothelial dysfunction (52). Moreover, the N207 glycosylation site of NS1 is highly conserved and is required by multiple flaviviruses (e.g., DENV, ZIKV and WNV) to trigger endothelial hyperpermeability (52). In summary, these data demonstrated that the N207Q mutant abolishes clathrin-mediated internalization of different flavivirus NS1 in human endothelial cells, which is necessary to trigger EGL disruption and induce endothelial barrier dysfunction (52).

The potential connection between multiple flavivirus NS1 and viral pathogenesis has also been reported recently, as NS1 from DENV, ZIKV, WNV, JEV and YFV could induce endothelial hyperpermeability in a cell-type-dependent (e.g., human endothelial cells from lung, dermis, umbilical vein, brain, and liver) manner *in vitro* and cause tissue-specific vascular leakage *in vivo*, which reflected the disease tropism of each flavivirus (95). Mechanistically, flavivirus NS1 can bind to endothelial cells selectively, which leads to differential disruption of EGL, finally resulting in tissue-specific vascular leak.

A novel study identified the molecular determinants for flavivirus NS1 binding specificity *in vitro* and causing tissue tropism *in vivo (* 96). By using chimeric NS1 that exchanged the wing and β-ladder domains among multiple flavivirus NS1 (e.g., DENV, ZIKV and WNV), it was pointed out that the wing domain of NS1 engaged in inducing endothelial hyperpermeability and further identified a three-residue motif 91 to 93 (GDI) within the wing domain of DENV NS1 that acts as a key factor contributing to EGL dysfunction and vascular leak (96). In contrast to the highly variable GDI motif among flaviviruses, there is also a conserved motif 115 to 118 (WWG) in the flexible loop within the wing domain among flaviviruses, which is involved in endothelial cell binding (97). Thus, it is reasonable to point out that flavivirus NS1 contains conserved motifs to interact with endothelial cells and variable residues that are implicated in tissue specificity.

Following the hypothesis that flavivirus NS1 may play a distinct role *in vivo* and *in vitro*, further studies are needed to clarify the effects of other nonendothelial molecules on NS1-triggered vascular leak, which will provide new insight into flavivirus pathogenesis and generate new targets against flavivirus infections.

## 6 NS1 and mosquito vectors

Mosquitoes are natural vectors of mosquito-borne flaviviruses. The virus can propagate in various tissues of mosquitoes without producing dramatic pathological changes, indicating that mosquitoes have evolved their own unique antiviral mechanisms to regulate mosquito permissiveness to viral infection (98).

After infectious viral particles are taken into the mosquito midgut by an infectious blood meal, the particles must pass through the midgut barriers to launch effective infection before being disseminated throughout the mosquito’s body; subsequently, the virus overcomes the salivary gland barrier and is released into the saliva, finally being transmitted to the mammalian hosts (99).

Mosquito gut immunity is the first-line barrier to restrict virus infection (100). Some conserved innate immune pathways, including Toll, immune deficiency factor, Janus kinase-signal transduction and activators of transcription (JAK–STAT), as well as RNA interference and the gut microbiota, function together to inhibit virus replication in the mosquito gut epithelium (101–104).

However, to be effectively acquired by natural vectors, flaviviruses have also evolved their own strategies to conquer midgut immune barriers. Flavivirus NS1 can be secreted into the serum of infected hosts and plays a critical role in virion acquisition by vectors (54, 60, 105). Flavivirus NS1 helps the virus overcome the midgut immune barrier by suppressing the expression of immune genes related to reactive oxygen species and the JAK–STAT pathway, resulting in enhanced viral acquisition by mosquitos (54, 60). These data suggested that flavivirus NS1 might be a reasonable reason for flavivirus evolution to adapt to multiple hosts.

Mosquito vectors are indispensable components for flaviviruses to maintain their lifecycle in nature. Although our knowledge of the interactions between them has rapidly expanded, there are numerous puzzling questions that must be further explored. The mechanisms by which NS1 enhances viral infectivity during mosquito feeding should be a target for future investigation.

## 7 Conclusion

The global incidence of flavivirus infections has steadily increased over the past decades, representing a significant threat to public health worldwide. NS1 is among the key factors contributing to the severity of flavivirus disease. This multifunctional protein participates in disease development by comprising viral RC to participate in RNA replication, interaction with complement components, disrupting the EGL of endothelial cells, and increasing its acquisition by mosquito vectors. Although numerous studies have expanded our knowledge on flavivirus NS1 in viral replication, immune evasion, pathogenesis and interaction with natural hosts, there are many gaps in our knowledge concerning the molecular mechanisms by which NS1 functions in those aspects.

The major remaining challenge is to clarify more interactions between flavivirus NS1 and host factors, which will further clarify flavivirus pathogenesis and provide novel strategies for limiting viral transmission in nature.

## Author contributions

SeZ contributed to the design of the manuscript. SC and AC critically revised the manuscript. YH, ZW, MW, RJ, DZ, ML, XZ, QY, YW, ShZ, JH, SM, XO, QG, DS, LZ, and YY provided ideas contributing to the conception of this manuscript. All authors contributed to the article and approved the submitted version.
